# “Alcohol use and suicide in Lithuania: proximity shouting out loud”

**DOI:** 10.1192/j.eurpsy.2023.160

**Published:** 2023-07-19

**Authors:** D. Jokūbonis

**Affiliations:** ^1^Psychiatry, Lithuanian University of Health Sciences; ^2^Kaunas branch, Republican Center for Addictive Disorders, Kaunas, Lithuania

## Abstract

**Abstract:**

In the recent past, the standardized suicide mortality rate (SMR) in Lithuania was more than double the global SMR and nearly one and a half higher than in the EU. Herewith Lithuania has high male-to-female SMR ratios e.g., at 7.1 in 2016. Autopsy studies in Lithuania revealed that approximately two thirds of men and one third of women who died by suicide had a blood alcohol con-centration level above 0.04 g/dL. Although suicide is a complex phenomenon, heavy alcohol use has been considered as it’s important risk factor though the relationship was never systematically studied before in Lithuania. Experts have suggested that gender differences in excessive alcohol consumption can explain the gender disparity in suicide mortality and linked tackling the harmful use of alcohol as an opportunity for suicide prevention. Alcohol control policies may cause immediate effect on excessive alcohol consumption at both the population and individual-level and may be capable of impacting suicide mortality rates by altering alcohol use patterns at both domains. Implementation of alcohol taxation policy in Lithuania provided an opportunity to evaluate it‘s impact on suicide mortality in a country comparable to other high income countries with a comparable health care and medical education systems.

**Disclosure of Interest:**

None Declared

